# Catch-Up Screening to Improve Detection of Congenital Chagas Disease in a Nonendemic Setting

**DOI:** 10.4269/ajtmh.25-0656

**Published:** 2026-02-26

**Authors:** Anna Boté-Casamitjana, Joanna Martin, Jackie Touray, Natalie Elkheir, Laura Eve Nabarro, Sarah Eisen, David A. J Moore

**Affiliations:** ^1^ Hospital for Tropical Diseases, University College London Hospitals NHS Trust, London, United Kingdom;; ^2^ Children and Young People’s Division, University College London Hospitals NHS Foundation Trust, London, United Kingdom;; ^3^ Clinical Research Department, London School of Hygiene and Tropical Medicine, London, United Kingdom

## Abstract

Mother-to-child transmission of *Trypanosoma cruzi*, the parasitic cause of Chagas disease, occurs in 5–10% of affected pregnancies. Congenital infection is usually asymptomatic, so if maternal infection is not suspected, the affected newborn may go undetected. As detection and antiparasitic treatment in early life result in much higher cure rates than treatment later in life, it is important to explore opportunities for catch-up testing of children born to women found to have *T. cruzi* infection. We piloted a multidisciplinary adult–pediatric disease referral pathway and developed a clinic to screen children of women diagnosed with Chagas disease. We evaluated the impact, acceptability, and patient experience of the pathway, including exploration of barriers to accessing prior testing. Of the 28 referred children, 23 (82%) attended the clinic, 22 (79%) were tested, and 1 child was diagnosed with congenital infection. The service was deemed effective and beneficial by both healthcare professionals and parents.

## INTRODUCTION

Chagas disease is caused by the protozoan parasite *Trypanosoma cruzi.* Endemic to rural areas of Latin America, global migration has resulted in hundreds of thousands of chronically infected people living in nonendemic countries. Antiparasitic treatment with benznidazole or nifurtimox is most effective soon after infection and in children, preventing progression to chronic disease, but it is poorly tolerated and contraindicated in pregnancy.^1^^,^^2^ Chagas disease is transmitted via infected triatomine bugs in endemic areas but can also spread nonvectorially through vertical transmission, blood transfusions, and organ transplants.^3^^,^^4^

Mother-to-child transmission occurs in 5–10% of pregnancies, and untreated women of childbearing age can transmit the infection during each pregnancy.^5^ The World Health Organization and the Pan American Health Organization recommend antenatal screening. Such programs are limited to local or regional levels in nonendemic countries.^6^^–^^9^ In the United Kingdom, the Migrant Health Guide recommends screening migrants from endemic areas, particularly pregnant women and other women of reproductive age.^10^ However, there is no formal antenatal screening program nor is there consensus on how best to do this, resulting in low uptake and delayed diagnoses.^11^

The Hospital for Tropical Diseases (HTD), a tertiary referral center, collaborates with the United Kingdom Chagas Hub, which organizes community screening events.^11^ These events have identified numerous new cases of Chagas disease in London, many of whom are women of childbearing age. During initial appointments, women are offered treatment, informed about the risks of vertical transmission, and advised to have their children and relatives at risk tested or referred through their general practitioners (GPs). Pregnant women are counseled on the need for neonatal testing, and a birth plan is made to arrange infant screening at the place of delivery.

Arranging children’s testing via GPs has proven challenging. This is likely due to a lack of awareness of the disease in primary care and difficulties accessing the necessary serological tests. This results in many children not accessing screening.^12^ Barriers to testing may also stem from healthcare access challenges related to language, cultural issues, immigration status, and financial constraints.^13^

As a result, we developed and evaluated a streamlined referral pathway combining adult and pediatric services to screen, manage, and follow up children at risk. The service was offered to women diagnosed with Chagas disease who attended the Adult Infectious Diseases clinics and had children at risk of mother-to-child transmission. These women were contacted by phone by an infectious disease doctor fluent in Spanish and provided with information about the new referral pathway. Children identified as needing screening were referred to the pediatric infectious diseases team. Appointment letters were translated into Spanish and Portuguese.

Following an inclusion health approach, up to three appointments were offered to children who did not attend the initial visit, with results shared with patients, GPs, and the adult team. This process was coordinated by the pediatrics clinic coordinator and supported by the infectious diseases and inclusion health clinical nurse specialist.

Numbers of referrals, attendances, completed tests, and outcomes were audited. Maternal experiences, including barriers to before testing, were explored via semistructured interviews conducted in Spanish by a native-speaking doctor. The service evaluation did not require ethical approval and was registered according to local governance processes.

Twenty-one women were identified within the adult service. Two women had children aged 18 or older, who were offered appointments through the adult service, and 3 women had children who had already been screened. This resulted in 16 women and 28 children eligible for the referral pathway.

There were no pregnant women in this cohort; however, during the contact process, one infant under 3 months of age was identified. This infant was born to a mother seen in our clinic prenatally and was contacted because she had an older child born before her diagnosis. Although a plan had been established for her maternity services to arrange local neonatal Chagas disease testing,^14^ this was not followed correctly. As a result, the infant was offered further testing at our center.

Median maternal age was 41.5 years (interquartile range [IQR] 39–46.3), and most women (94%; 15/16) were born in Bolivia. The median age of the children was 10.5 years (IQR 8–15), and 50% were female. Most children were born in Chagas nonendemic countries, with 50% (14/28) born in Spain, 43% (12/28) in the United Kingdom, and only 7% (2/28) in Bolivia.

**Figure 1.**
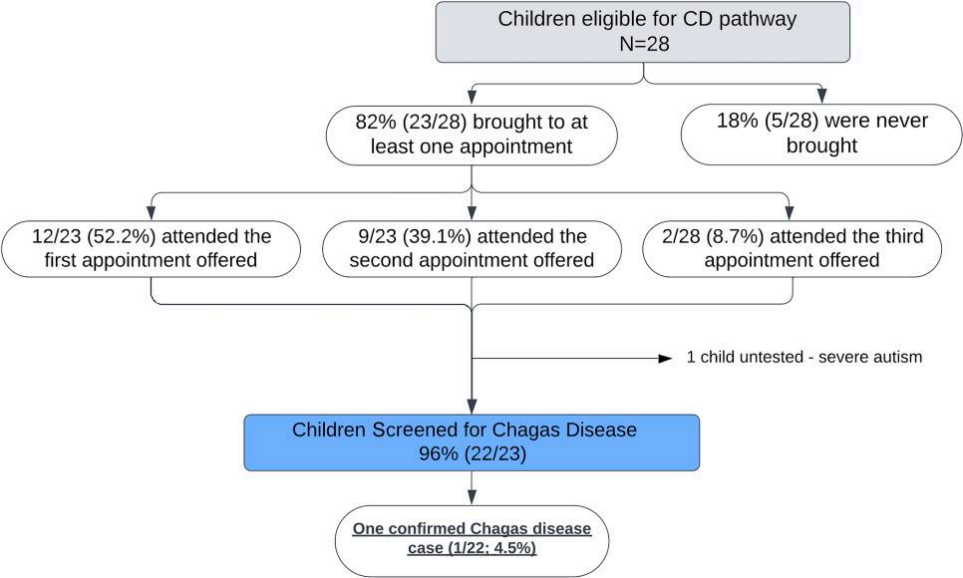
Flowchart illustrating adult–pediatric referral pathway for screening children at risk of congenital Chagas. CD = Chagas disease.

In the 6 months after the initial contact, 28 children were booked for testing. Of these, 23 children (82%) were brought to at least one appointment (Figure 1).

Testing for Chagas disease was offered to all 23 children. One child did not receive testing due to severe autism, requiring general anesthetics for phlebotomy procedures, but a plan was established for opportunistic future testing. Serum samples were screened using a commercial IgG recombinant antigen-based ELISA assay (Chagatest ELISA Recombinante v.4.0, Wiener Laboratory, Rosario, Argentina). When positive, results were confirmed by an in-house, fully validated indirect fluorescent antibody test.^15^

Among the 22 children tested, a 16-year-old child born and raised in the United Kingdom to a Bolivian mother was found to have positive *T. cruzi* serology and was diagnosed with indeterminate congenital Chagas disease. Because benznidazole was unavailable in the United Kingdom at the time (stock issues), treatment with nifurtimox was started. The child completed treatment despite experiencing significant side effects.

Of 16 women whose children had been referred for testing, 15 were approached by phone to schedule an interview. One participant was deceased due to an unrelated cause. Two women were uncontactable, and 13 women agreed to participate. Ultimately, 10 interviews were completed. The women interviewed had been engaged with our service for a median of 2 years (IQR 1–2). A mixed inductive–deductive approach was used to analyze the qualitative data. Key themes were identified beforehand to guide the interviews, and the transcripts were then manually coded to allow additional subthemes to emerge.

Most participants described their experiences with the pediatric Chagas disease clinic as excellent, emphasizing the professionalism of the staff and the individualized attention provided to their children. Many participants were pleased with how the department was organized and appreciated that appointments were communicated in Spanish.

Many mothers were concerned about the risk of transmission of Chagas disease to their children and were aware of the higher treatment tolerance and effectiveness in children, with some stating concerns about the potential impact on their children’s long-term well-being (Table 1, Quotation 1).

**Table 1:** Quotations

Quote No.	Theme or Topic	Quotation
Q1	Perception of risk	“I was worried, yes, because I’m aware of how this disease can react when it is active in the body. I have close uncles who have had to suffer from the disease and have not received enough support, um... I’m talking about medical support, so I know what the aftereffects and consequences are. So obviously, I worry about my children much more than I might worry about myself.”
Q2 and Q3	Challenges in accessing and maintaining care due to migration and health system barriers	“My only hope was to schedule an appointment with a doctor in Spain and go... to organize a trip and take my children to Spain for the test. I knew it was urgent, but, you know, economically, sometimes one cannot... because just imagine traveling with a family of four children to Spain, arranging everything, and not always having a place to stay... it’s very difficult.” “My younger daughter was born in Spain. I brought her here to London when she was nine months old. When she was born, they did a test after a month, and it came back positive. My older daughter also tested positive, but they said the antibodies weren’t active… I don’t know… I couldn’t really explain it properly, I didn’t understand it at the time. They told me to go back, but since then I haven’t gone back… by the following year we were already here in London.”
Q4 & Q5	Perceived barriers to primary care testing	“You talk to them about Chagas, and they say ‘what’s that?’ Yes, I think it’s a lack of knowledge, because I told them that I had already gone to the GP a while ago to try to get my children tested, and they said they would look into it, but that was it. I asked them because my daughter was having a lot of difficulties—everything in her tests came back fine, and I kept thinking, something’s wrong… maybe she has Chagas… She would tell me her heart hurt… she would walk a little and get tired.” “I had many health problems, and I reached a point where I could hardly breathe. I don’t know how I thought of Chagas, and I told her [the GP] that I wanted to get tested. She said, ‘No, no, not here; that’s not here.’ I insisted, saying, ‘Look, I’m from Bolivia, and my parents had Chagas. Now I find out that some of my siblings have also had Chagas... and it’s possible...’ But the doctor kept telling me no, that it wasn’t here. I told her that even though I had been here for a while, I didn’t know, and I wanted to take the test. Well, I insisted, and she finally said, ‘Okay, if you want to, do it.’ After the test, she told me that I tested positive... and thanks to that test, my health has improved... I’ve improved a lot, so...”
Q6	Time, cost, and travel challenges	“They don’t want to give my eldest daughter permission. They say we should schedule appointments when they are on vacation. If it’s not important or urgent, we shouldn’t make appointments, the school told me. I told them once (about the Chagas test), but usually, they don’t know what that is.”
Q7	Community demand and awareness of Chagas testing	“I have met many people who are looking for the clinic because I have mentioned the topic of Chagas to them, and they say, ‘Oh, please, can you give me the address? Because I have had Chagas for a long time, and I didn’t know that they treated it here...’”

GP = general practitioner; Q = quote.

Several mothers commented on challenges experienced due to their migration status. Even when children had been prescreened in the past, mothers often did not understand the significance of the results, and follow-up appointments were frequently missed due to relocation. Participants also reported difficulties in navigating the National Health Service. Sometimes, they found it easier to return to other countries to continue receiving care (Table 1, Quotations 2 and 3).

Many who pursued primary-care testing in the United Kingdom reported difficulties from lack of GP awareness of the disease. Others explained they had not requested testing through primary care because they felt their GPs might be unfamiliar with Chagas disease (Table 1, Quotations 4 and 5).

With regard to hospital testing, cost (time and financial) was recognized as a potential barrier. Many highlighted traveling long distances, cost, and unreliable train services as challenges. Women also reported difficulties obtaining permission from employers for time off and losing income to attend the clinics, and further difficulties with schools when trying to arrange children’s medical appointments. Schools were often hesitant to grant permission for absences (Table 1, Quotation 6).

Some participants encountered other challenges, such as missed or late appointment letters or logistical issues (e.g., difficulties coordinating Chagas disease clinics with other medical appointments).

During interviews, most women mentioned a strong demand for Chagas disease testing within the Latin American community in London. Participants wanted to discuss the available testing pathways for their friends and relatives, explaining that many desired testing. They also explained that many recently migrated individuals are unaware of the Chagas disease health services available in London (Table 1, Quotation 7).

In response to these challenges of the Latin American community accessing timely Chagas disease screening for their children in London, we established a combined adult–pediatric infection service pilot to identify and screen for congenital disease. This service succeeded in high engagement and satisfaction and delivered effective screening to eligible children. We diagnosed one case of congenital Chagas in a 16-year-old child born in a nonendemic country, enabling treatment and consequent reduction in risk of complications. This late-diagnosed case and number of unscreened children identified through this service contributed to the case for effective and consistent national implementation of screening programs for migrants from endemic countries entering the United Kingdom

Most women in our cohort were Bolivian, reflecting a higher prevalence of infection in this country, and the longstanding engagement of Bolivian communities with community-based screening initiatives in London. This also mirrors the demographic pattern observed in the HTD adult Chagas service, in which Bolivians constitute the majority diagnosed. However, this may not fully represent the wider Latin American community in the United Kingdom, and an unmeasured burden of disease among other migrant groups is likely. Therefore, ongoing efforts by organizations such as the Indoamerican Refugee and Migrant Organization aim to broaden access to testing.

Barriers to accessing screening identified in our cohort are consistent with previous data.^16^^–^^18^ By following the proposed multidimensional framework by Forsyth et al.,^17^ the main barriers to healthcare access identified can be classified into four groups: 1) structural barriers due to limited flexibility to attend appointments due to work-related or transportation issues, 2) psychosocial barriers as a result of language barriers and insufficient social support, 3) systemic barriers related to lack of awareness about the disease, and 4) clinical barriers as a result of a lack of access to *T. cruzi* serology in some settings. Despite this, the women engaged in our service demonstrated a high level of knowledge about Chagas disease. This may reflect the sustained engagement with the adult Chagas Clinic, where patients receive counseling and education about the disease and its implications for their children, which may contrast with findings from other studies in migrant populations.^19^

Given the success of the pilot and the high levels of engagement and satisfaction among patients, we will maintain the pathway developed during this project and intend to upscale the model we have developed with a robust evaluation and codevelopment with those within the Latin American community. The service has now expanded to include direct referral pathways into our pediatric department for centers across the United Kingdom caring for adults with Chagas disease, facilitated through our national Chagas multidisciplinary team. In parallel, we will continue to strengthen inclusion health processes within these clinics and continue to develop community-based programs to raise awareness of the condition and the need for screening. Ongoing work will also include producing educational leaflets for schools and exploring modifications to service delivery to improve access and engagement. Ultimately, systematic national screening for Chagas disease in target populations in the United Kingdom is needed to ensure that all at-risk individuals are identified and tested.

This pilot highlights the importance of testing all women of childbearing age and their children with robust and efficient multidisciplinary pathways with bespoke adaptations to promote an inclusive health approach in communities experiencing health inequalities. However, we recognize that the level of support required per patient may limit generalizability and feasibility in other settings.

## Supplemental Materials

10.4269/ajtmh.25-0656 Supplemental Materials
