# Supplementary material for: Catch-Up Screening to Improve Detection of Congenital Chagas Disease in a Nonendemic Setting
Source: Am J Trop Med Hyg. 2026 Feb 26;114(4):716–9. doi: 10.4269/ajtmh.25-0656 (PMC13045532; doi:10.4269/ajtmh.25-0656)
Supplement: Supplemental Materials [file tpmd250656.SD1.pdf]

Annex 1: Service Evaluation

Experience at the adult Chagas clinic

1. How many years have you been attending the Chagas clinic at UCLH?
2. On a scale of 1 to 10, what rating would you give the service you have received?
3. Have you experienced any difficulties? (e.g., language barrier, not understanding the information given to you...)
4. What positive aspects would you highlight?
5. How important is it to you that your doctor speaks Spanish?
    a. Very important
    b. Important
    c. I appreciate it, but it wouldn't be necessary
    d. Not important
6. Do you think we could do something specific to improve our service?
7. What do you think is the reason for this happening?
8. If this has also happened to you, we would like to hear your reasons and your own experience.
9. If you have ever missed an appointment, have you called or informed the hospital to change the date and time?
10. Currently, our Chagas clinic operates only one day a week in the morning (9-1pm) and another day in the afternoon (3-5pm). From your experience, do you have any suggestions on improving the service so that everyone who needs it can attend the visits?
11. We are evaluating the following options. Which would better suit your needs and those of other people attending the Chagas clinic?
    a. Access a nurse-led Chagas clinic every day of the week without having to make a prior appointment.
    b. Opening a clinic on weekends.
    c. Opening an evening clinic
    d. Opening a clinic during special dates (for example, school or summer holidays like half term).

Experience at the Chagas clinic for children

1. Before I contacted you a few months ago, were you aware that your children needed a Chagas test? If so, how did you know?
2. Do you think you were given enough information to understand why your children needed the test?
3. Is it something that worried you? Do you think it is something important or urgent?
4. Have you tried to have them tested previously? What problems did you encounter/why didn't it happen?
5. What has been your experience attending the paediatric clinic to have your children tested?
6. Did you receive the confirmation letter for the appointment in Spanish? Was it helpful?
7. Did you get any further reminders? (i.e. text messages)
8. How easy was it for you to attend the clinics? Could you afford it?

9. According to our records, you were unable to attend the first appointment offered to you. Would you like to tell me the reason for not attending?
10. Did you receive the results of your child's test?
11. Do you think there is anything we can improve?
12. We are evaluating the best way to organise tests for children who need them from now on. What do you think would be the best way to test the children of mothers with Chagas?
    a. Place and time of year/day?
    b. Testing in adult clinics
13. Would you bring any future children to the clinic to be tested?

As we discussed before, do you think, for example, opening clinics on Saturdays, having the flexibility to go to the clinic any day of the week, or being able to do rapid tests in the community would be a better option than the current one?
